# Supplementary figures and images for: Identification of transporters essential for survival of Leishmania promastigotes in the digestive tract of sand flies
Source: PLoS Pathog. 2026 Mar 16;22(3):e1014049. doi: 10.1371/journal.ppat.1014049 (PMC13004518; doi:10.1371/journal.ppat.1014049)

A

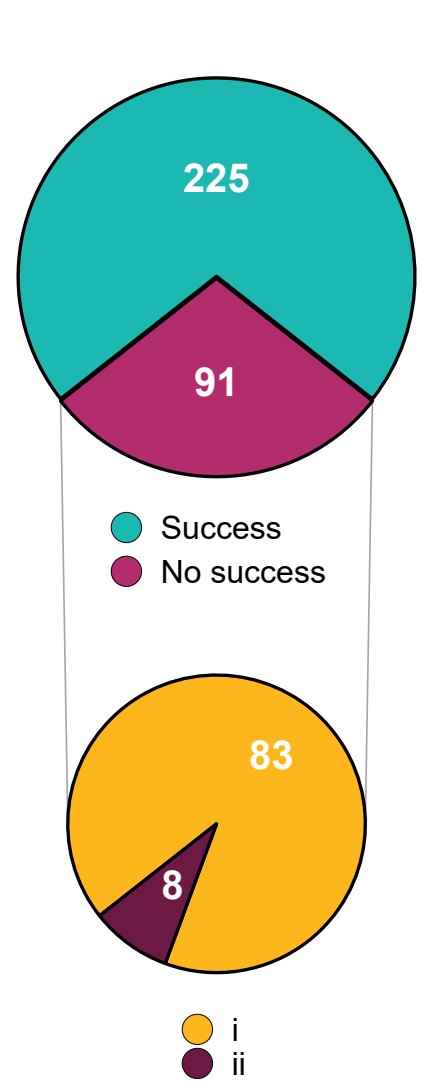

B

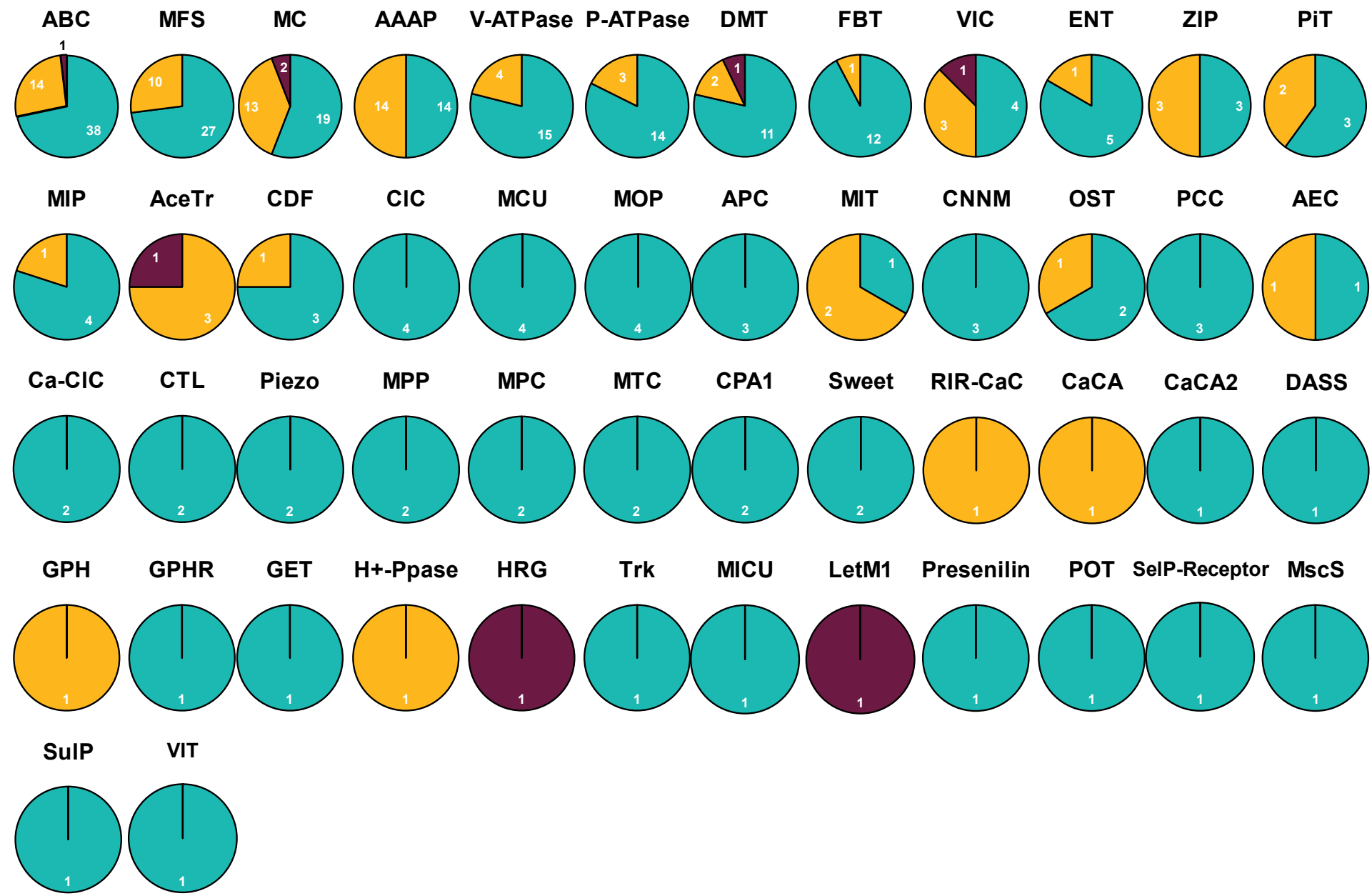

Supplementary Figure 1

Supplement: S1 Fig — (A) Top, pie-charts showing the numbers of successful gene deletions (cyan) and non-successful deletion attempts (magenta), across two independent screens (44 and this study). Bottom, break-down of non-successful deletions attempts into two sub-categories: (i) Double drug-resistant populations where ORF is still detected (or PCR inconclusive) (yellow); (ii) Attempts where no drug resistant populations were ever recovered, or populations where resistant cells could only be recovered with single drug selection and ORF was still detected (dark pink). (B) Summary of gene deletion results separated into TCDB families (S3 Table); colours as for A. (PDF) [file ppat.1014049.s001.pdf]

**A**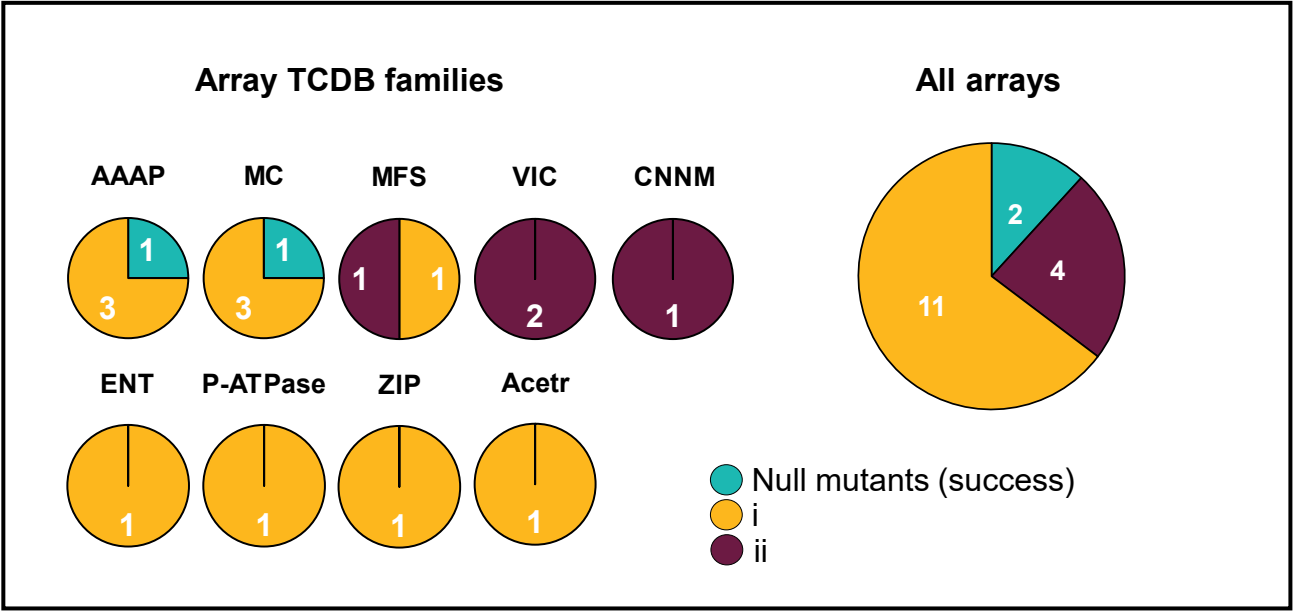**B**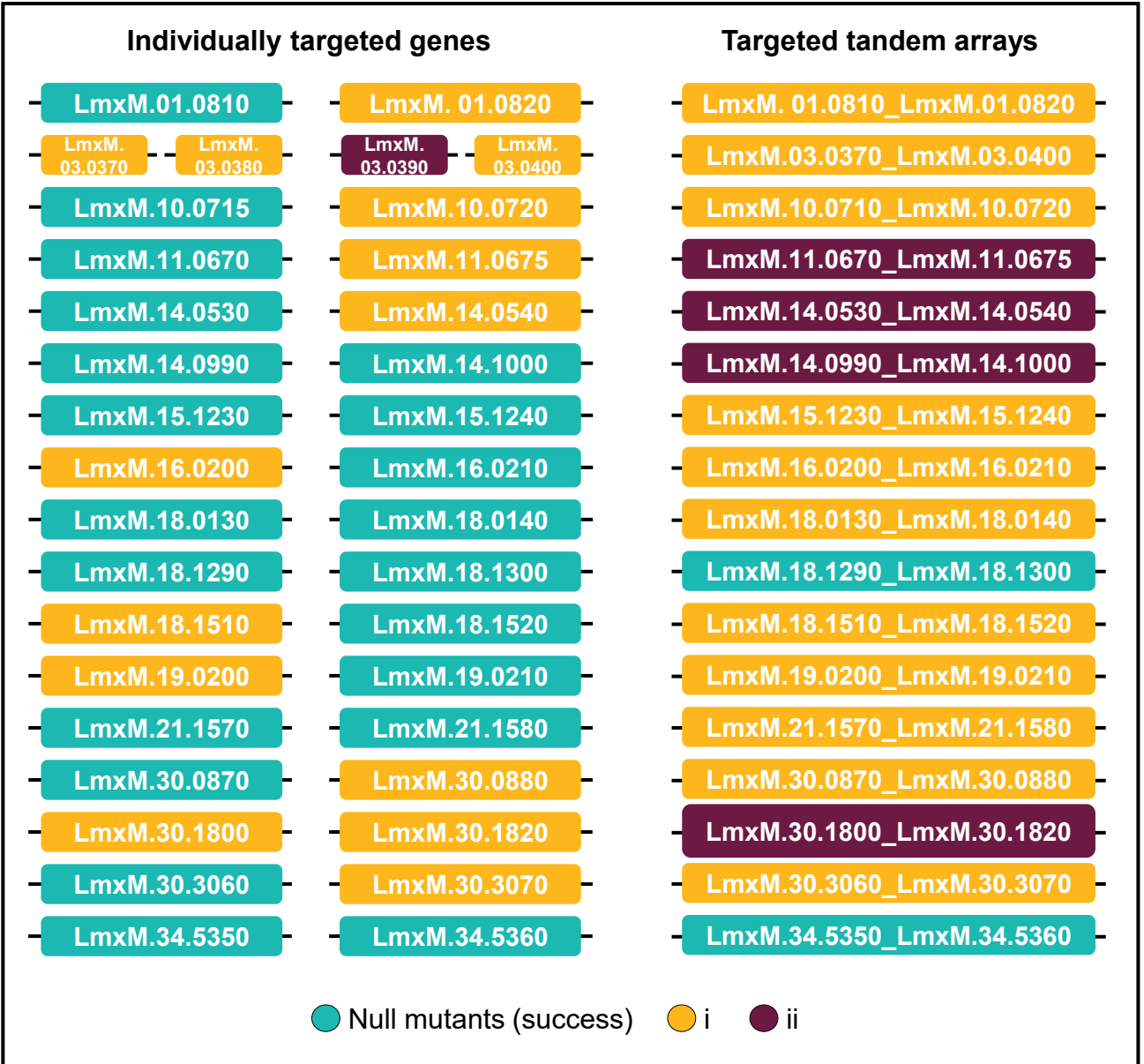

Supplementary Figure 3

Supplement: S3 Fig — (A) Pie-charts summarising gene deletion results separated into TCDB families (left) and in total (right) (S3 Table). (B) Overview of all genotypes for single genes and whole targeted arrays, coloured in four main categories (as in S1 Fig). (PDF) [file ppat.1014049.s003.pdf]

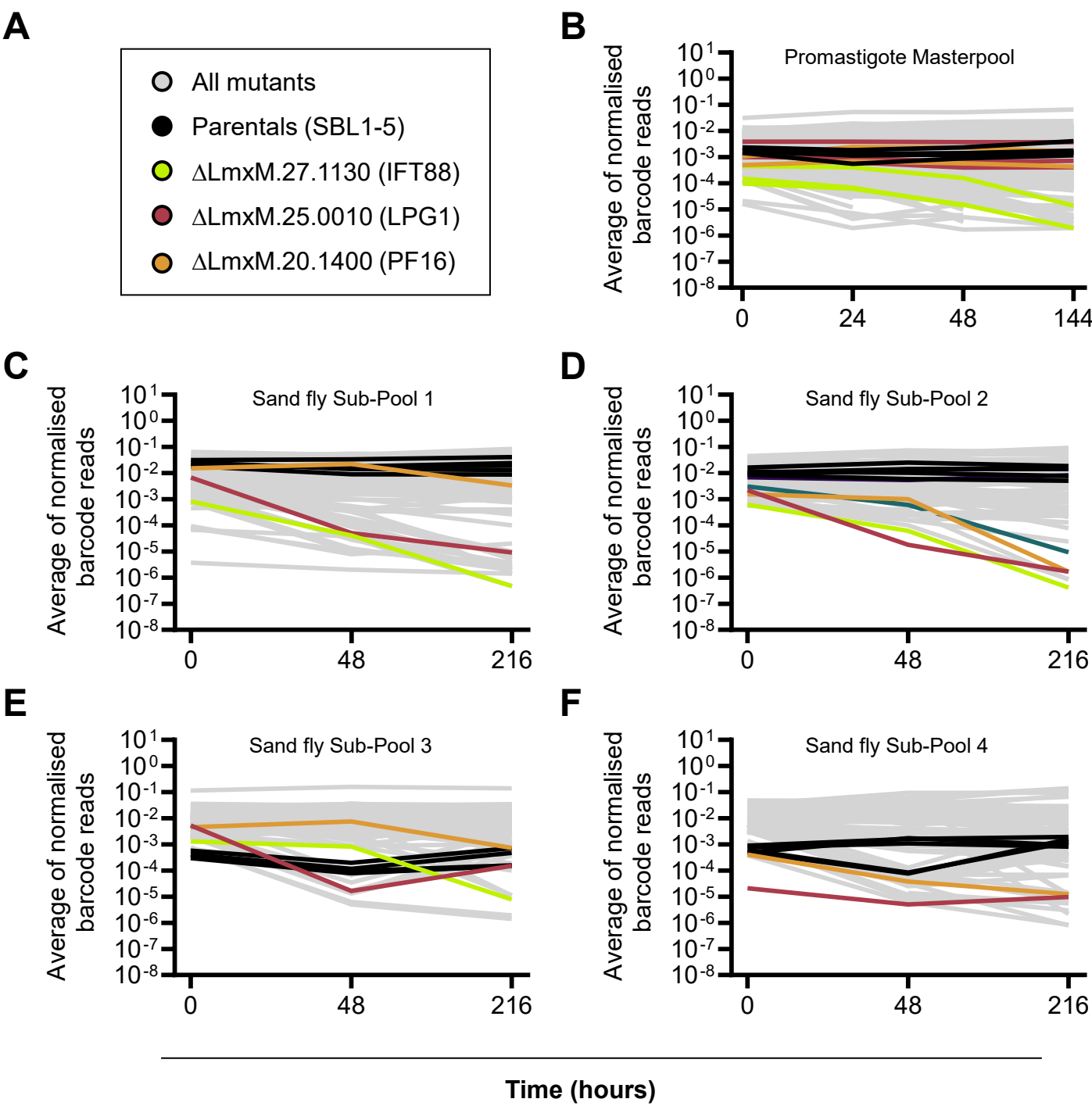

Supplementary Figure 4

Supplement: S4 Fig — (A) Plot legends. (B-F) Trajectories of the average of reads for all mutant barcodes in all conditions studied in this report, normalized to total reads for the experiment, reflecting the range of relative barcode proportions for the different mutants in each pool. (PDF) [file ppat.1014049.s004.pdf]

**A**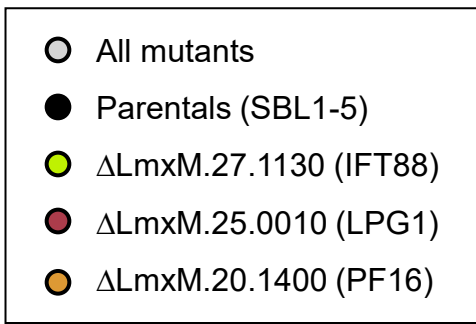**B**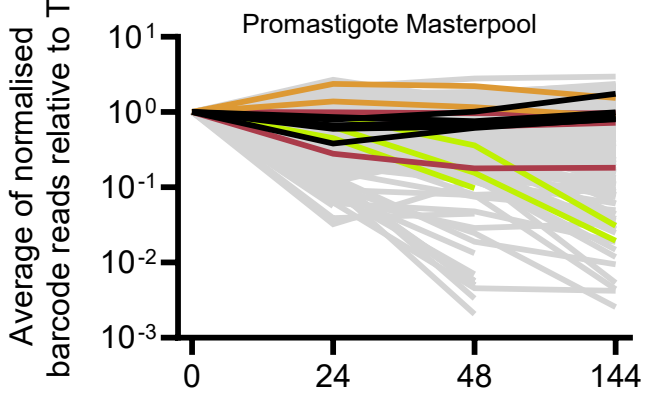**C**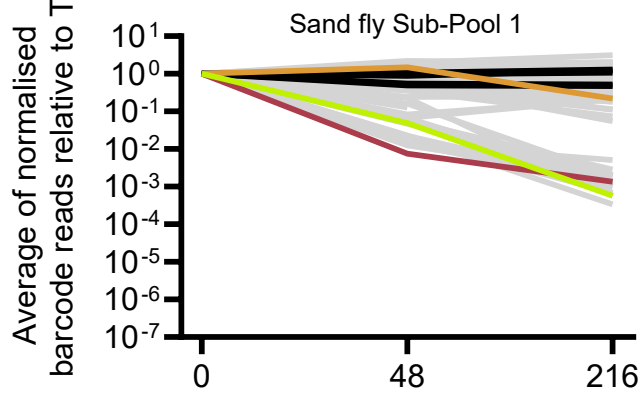**D**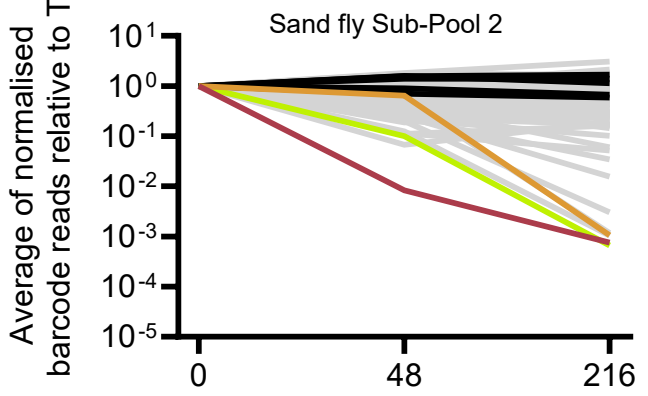**E**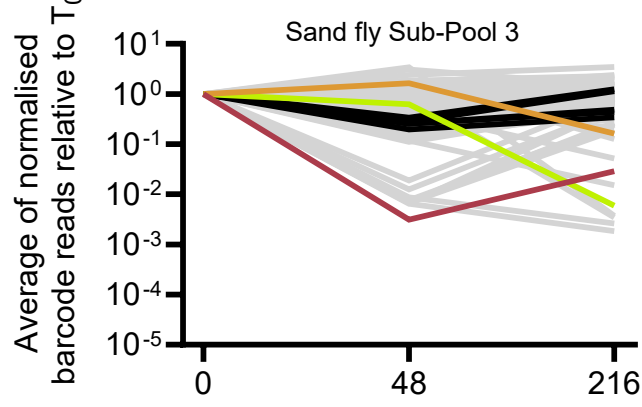**F**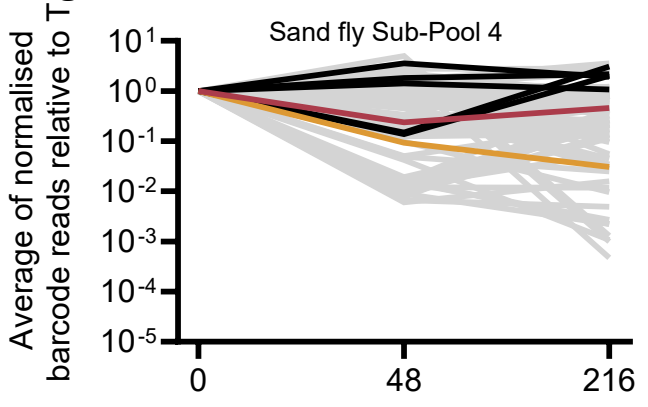

Time (hours)

Supplementary Figure 5

Supplement: S5 Fig — (A) Plot legends. (B-F) Trajectories of the average of barcode reads for all mutants in all conditions studied in this report, normalized to total reads for the experiment and relative to time-point “0 hours” (T0). (PDF) [file ppat.1014049.s005.pdf]

A

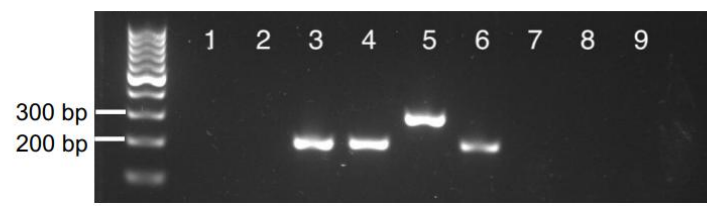

B

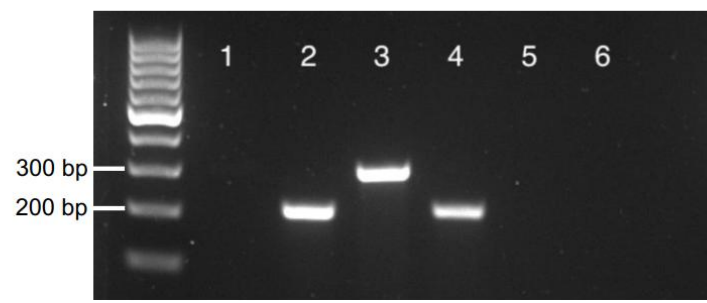

C

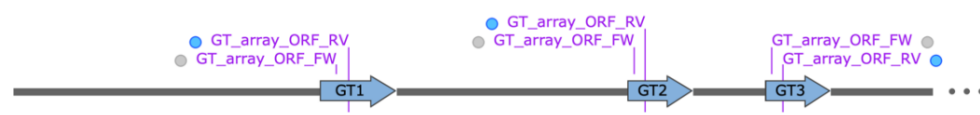

D

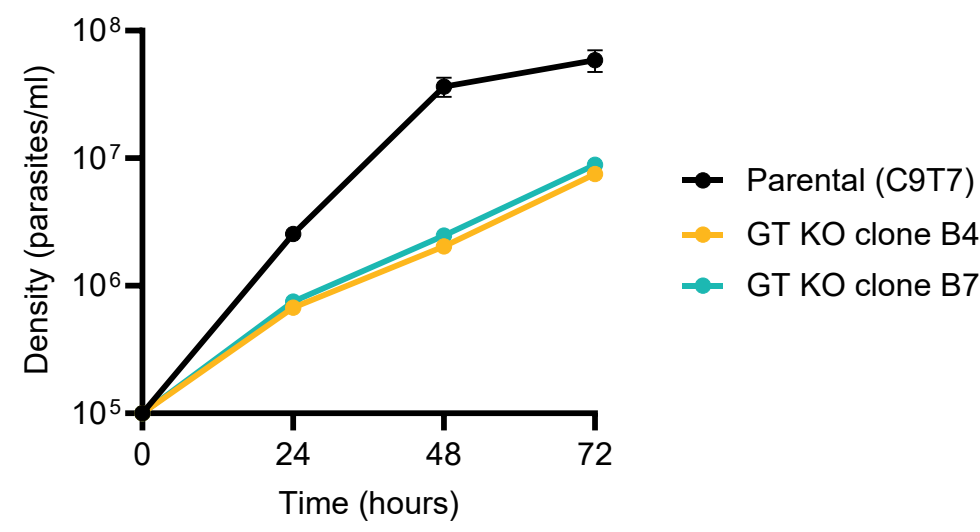

E

| Time period (hours) | Parental (C9T7) | GT KO Clone B4 | GT KO Clone B7 |
|---------------------|-----------------|----------------|----------------|
| 0 – 24              | 5.14            | 8.73           | 8.24           |
| 0 – 48              | 5.64            | 11.05          | 10.37          |
| 24 – 48             | 6.24            | 15.05          | 13.99          |
| 48 – 72             | 34.77           | 12.70          | 13.03          |

Supplementary Figure 6

Supplement: S6 Fig — (A) Diagnostic PCR gel electrophoresis of GT gene array null mutant clones. Lane 1, clone B4 gDNA + GT_array_ORF primers; lane 2, clone B7 gDNA and GT_array_ORF primers; lane 3, clone B4 gDNA and PFR2 primers (positive control for gDNA); lane 4, clone B7 gDNA and PFR2 primers; lane 5, parental gDNA and GT_array_ORF primers; lane 6, parental gDNA and PFR2 primers; lanes 7–9, gDNA of clones B4, B7 and parental without addition of primers. (B) Lane 1, clone H1 and GT_array_ORF primers; lane 2, clone H1 and PFR2 primers; lane 3, parental gDNA and GT_array_ORF primers; lane 4, parental gDNA and PFR2 primers; lanes 5 and 6, negative controls (no gDNA). DNA ladder: GeneRuler 100 bp DNA ladder by Thermo Scientific (REF: SM0243). (C) Locations of primer binding sites for diagnostic PCR. The forward primer (GT_array_ORF_FW) is highlighted with grey circles and the reverse primer (GT_array_ORF_RV) in blue circles. Note that both FW and RV primers recognise all glucose transporter open reading frames (ORFs) in the array. (D) Growth profile of GT KO clones over 72 hours. Black, Parental cell line; Yellow, LmGT array KO clone B4; Turquoise, LmGT array KO clone B7. Data points show the average of three measurements. (E) Doubling times of cells, calculated from D. (PDF) [file ppat.1014049.s006.pdf]

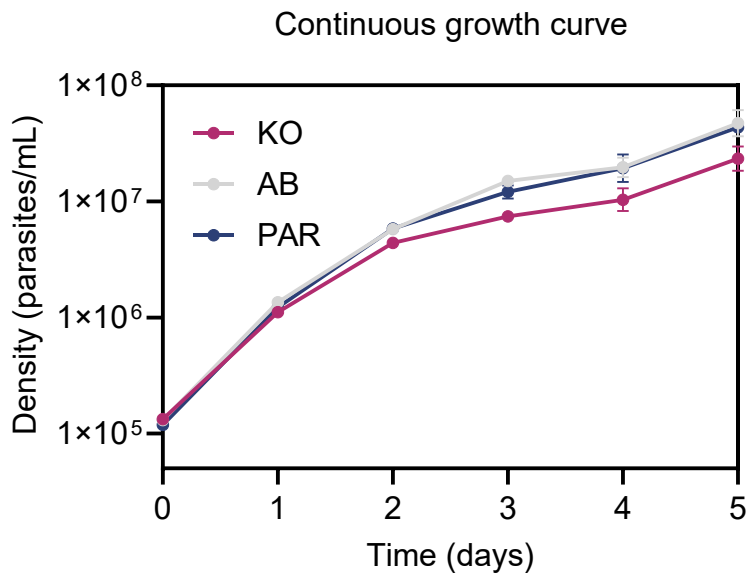

Supplementary Figure 7

Supplement: S7 Fig — Growth of parental (PAR, dark blue), V-ATPase V1E null mutant (KO, magenta) and V-ATPase V1E add-back (AB, grey) mutant promastigotes in vitro for 5 days of continuous growth. At each time point samples were collected for qPCR analysis (see Fig 4H). (PDF) [file ppat.1014049.s007.pdf]
