# Supplementary material for: Identification of transporters essential for survival of Leishmania promastigotes in the digestive tract of sand flies
Source: PLoS Pathog. 2026 Mar 16;22(3):e1014049. doi: 10.1371/journal.ppat.1014049 (PMC13004518; doi:10.1371/journal.ppat.1014049)
Supplement: S2 Fig — Results of diagnostic PCRs for genotypic validation of all new mutant cell lines reported in this study. (PDF) [file ppat.1014049.s002.pdf]

(A) Control PCR amplification of the blasticidin S acetyltransferase (Bla) encoding gene from mutant gDNA

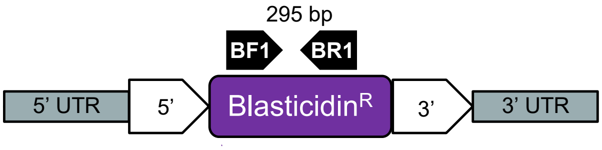

Group 1

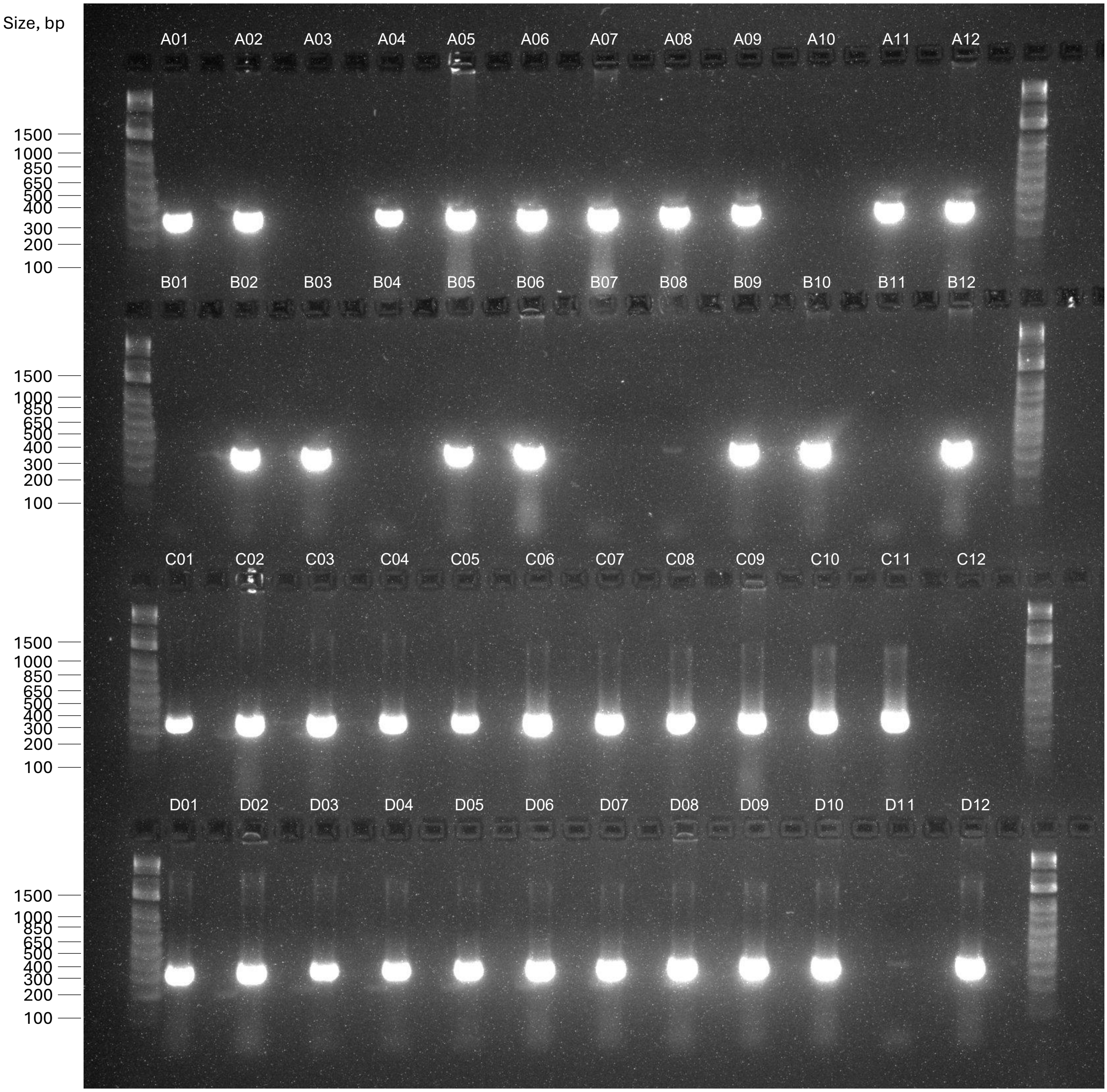

DNA ladder
1Kb Plus Dna Ladder, Ref. 10787018, ThermoFisher

Supplementary Figure 2

Group 1

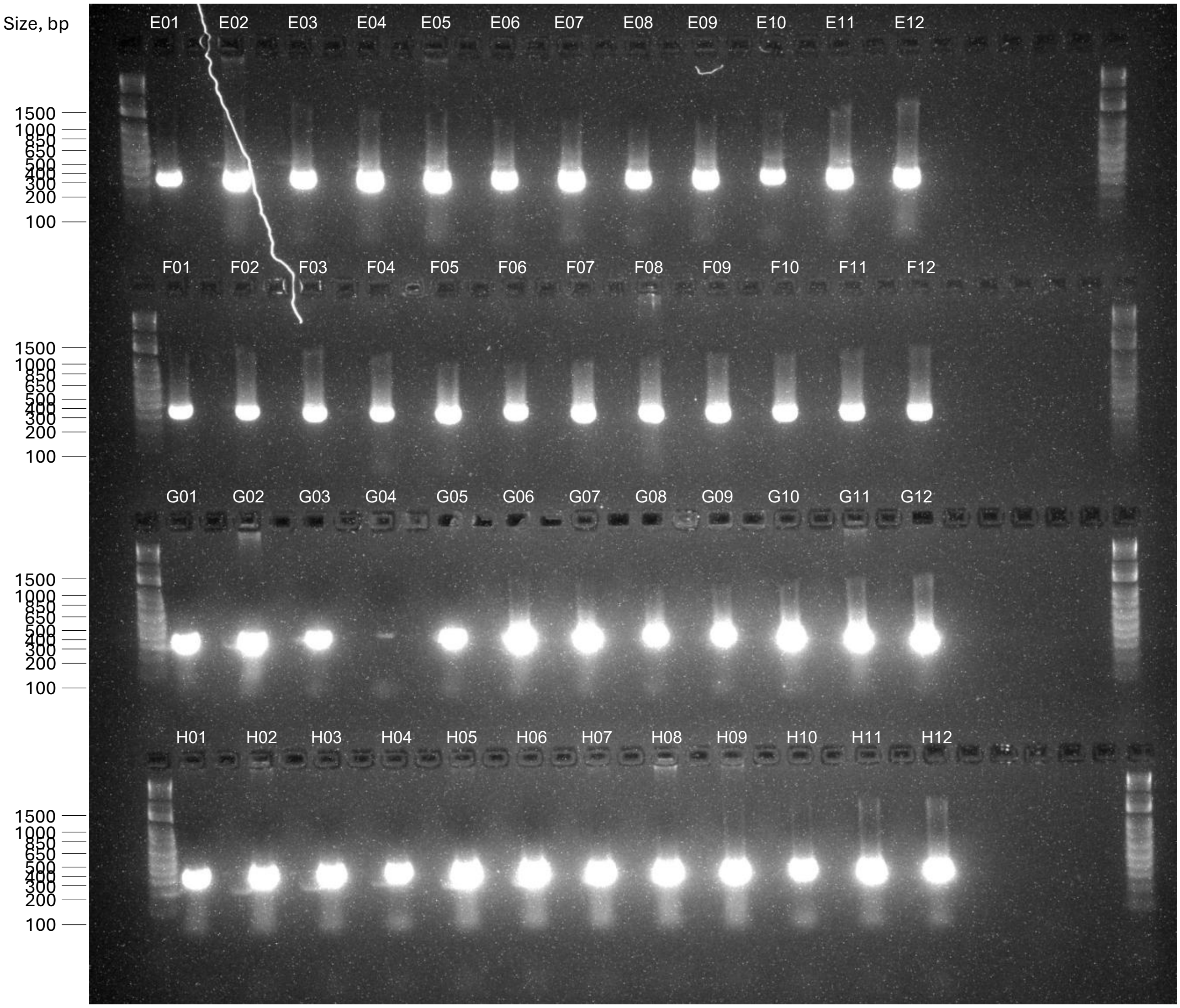

DNA ladder

1Kb Plus Dna Ladder, Ref. 10787018, ThermoFisher

Group 2

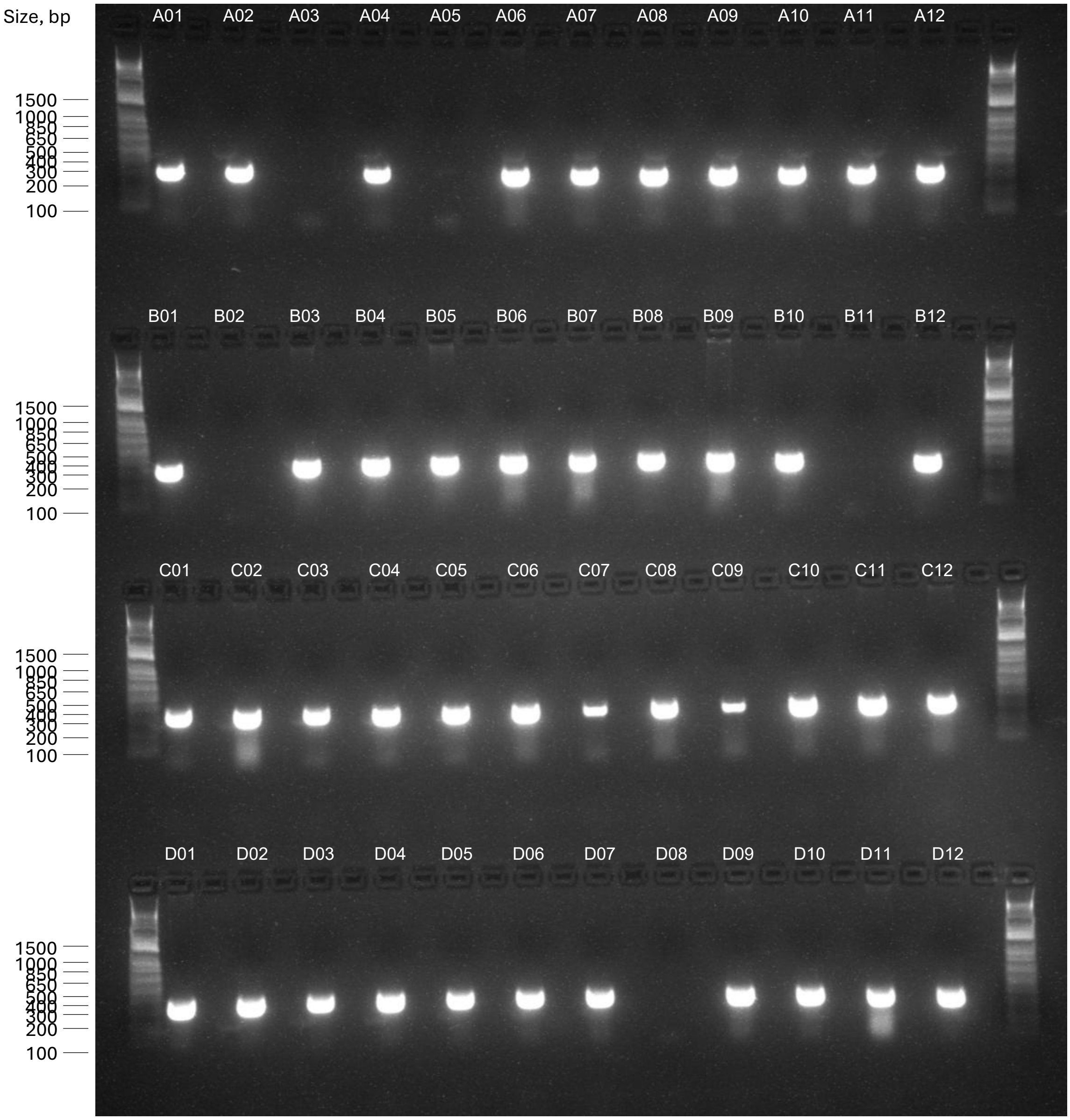

DNA ladder
1Kb Plus Dna Ladder, Ref. 10787018, ThermoFisher

Group 2

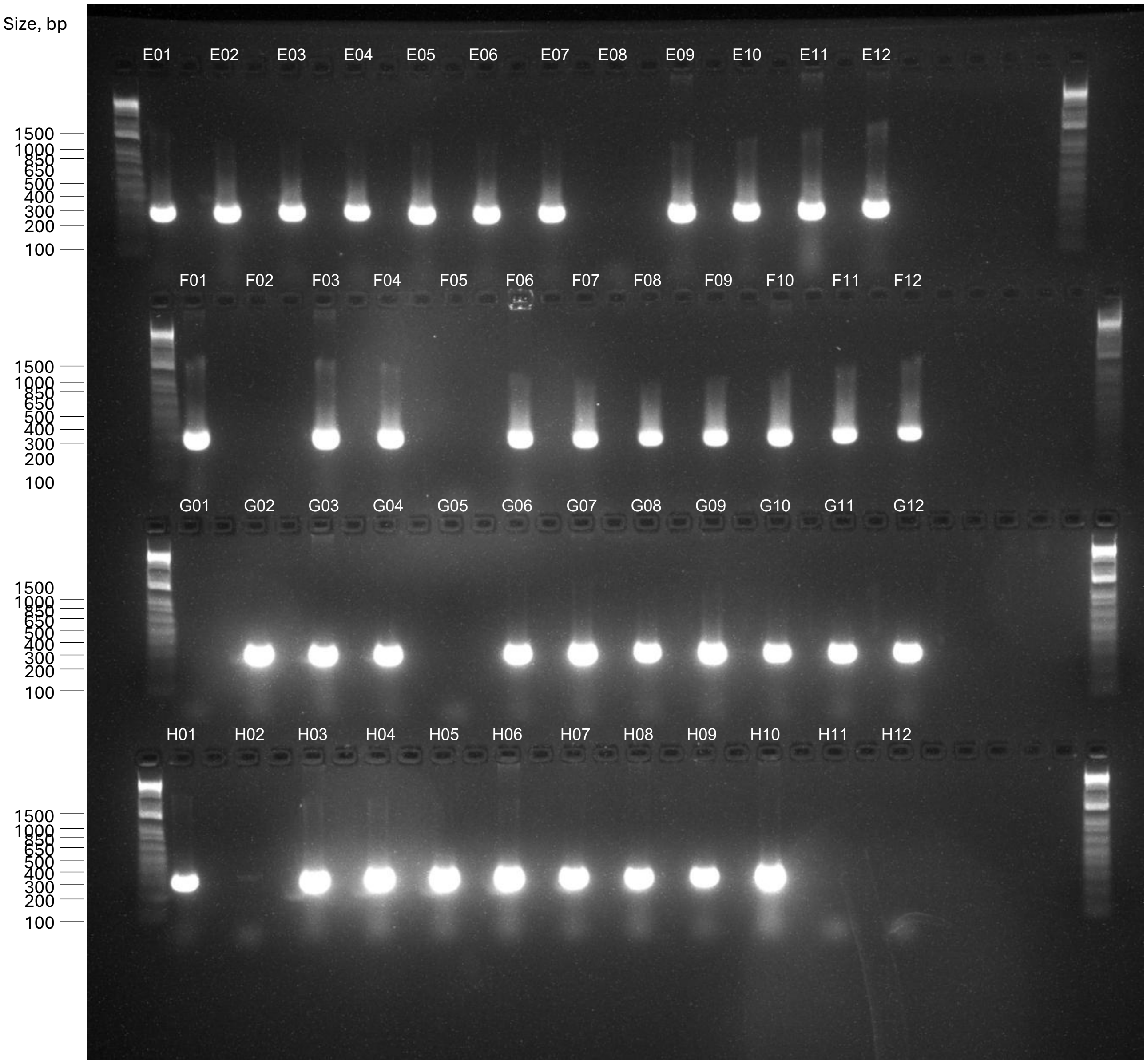

DNA ladder

1Kb Plus Dna Ladder, Ref. 10787018, ThermoFisher

Group 3

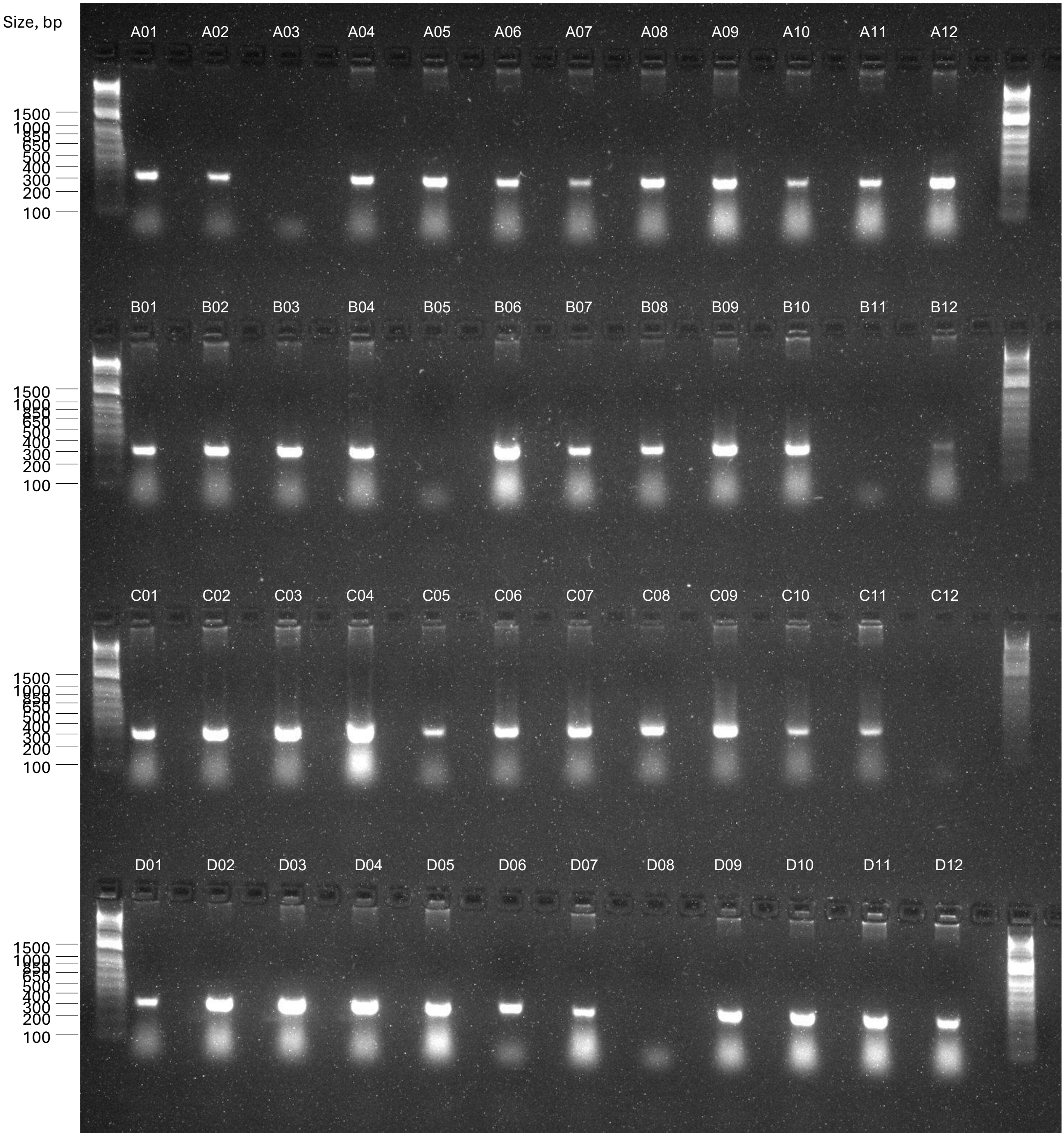

DNA ladder

1Kb Plus Dna Ladder, Ref. 10787018, ThermoFisher

Group 3

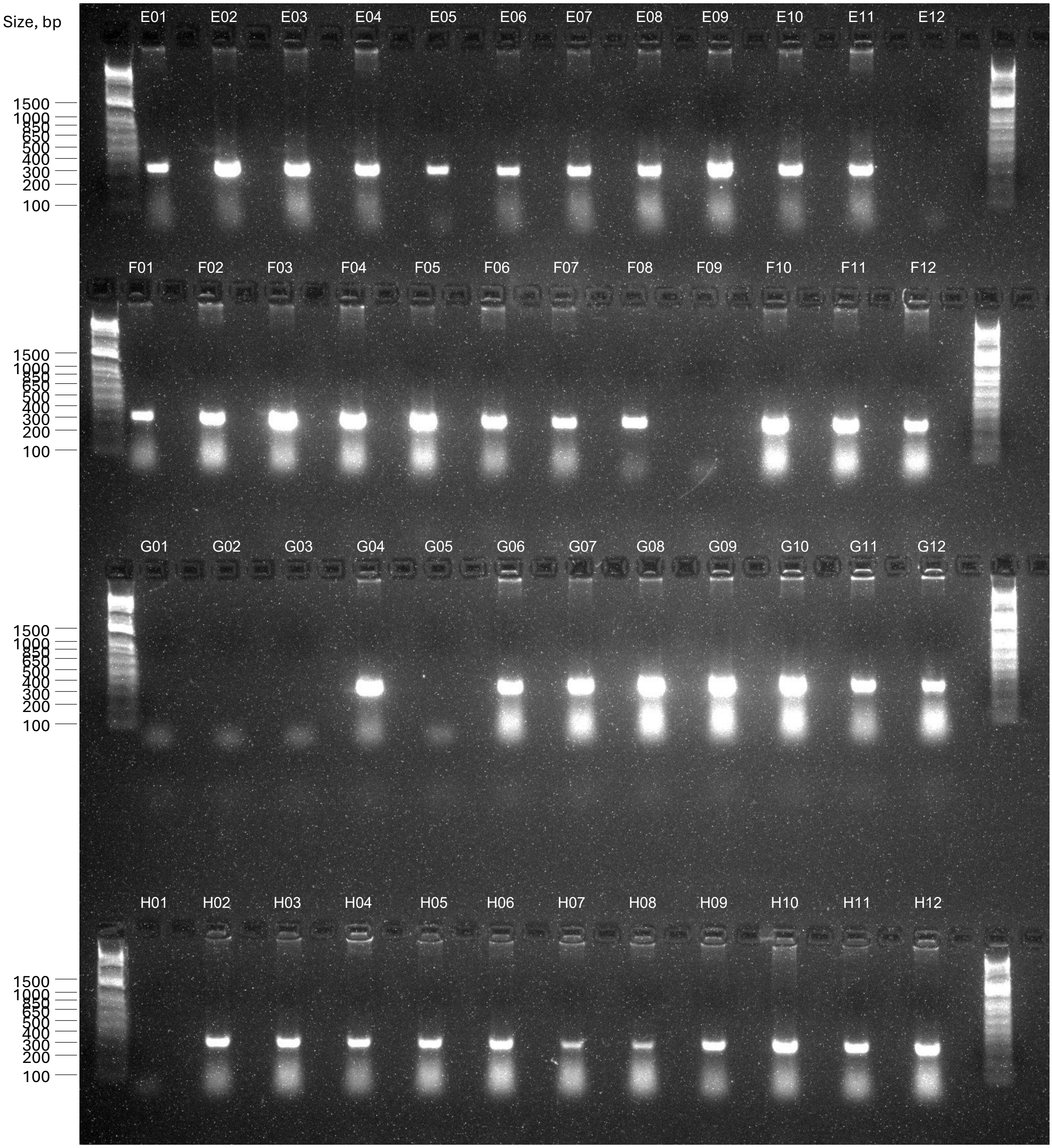

DNA ladder

1Kb Plus Dna Ladder, Ref. 10787018, ThermoFisher

Group 4

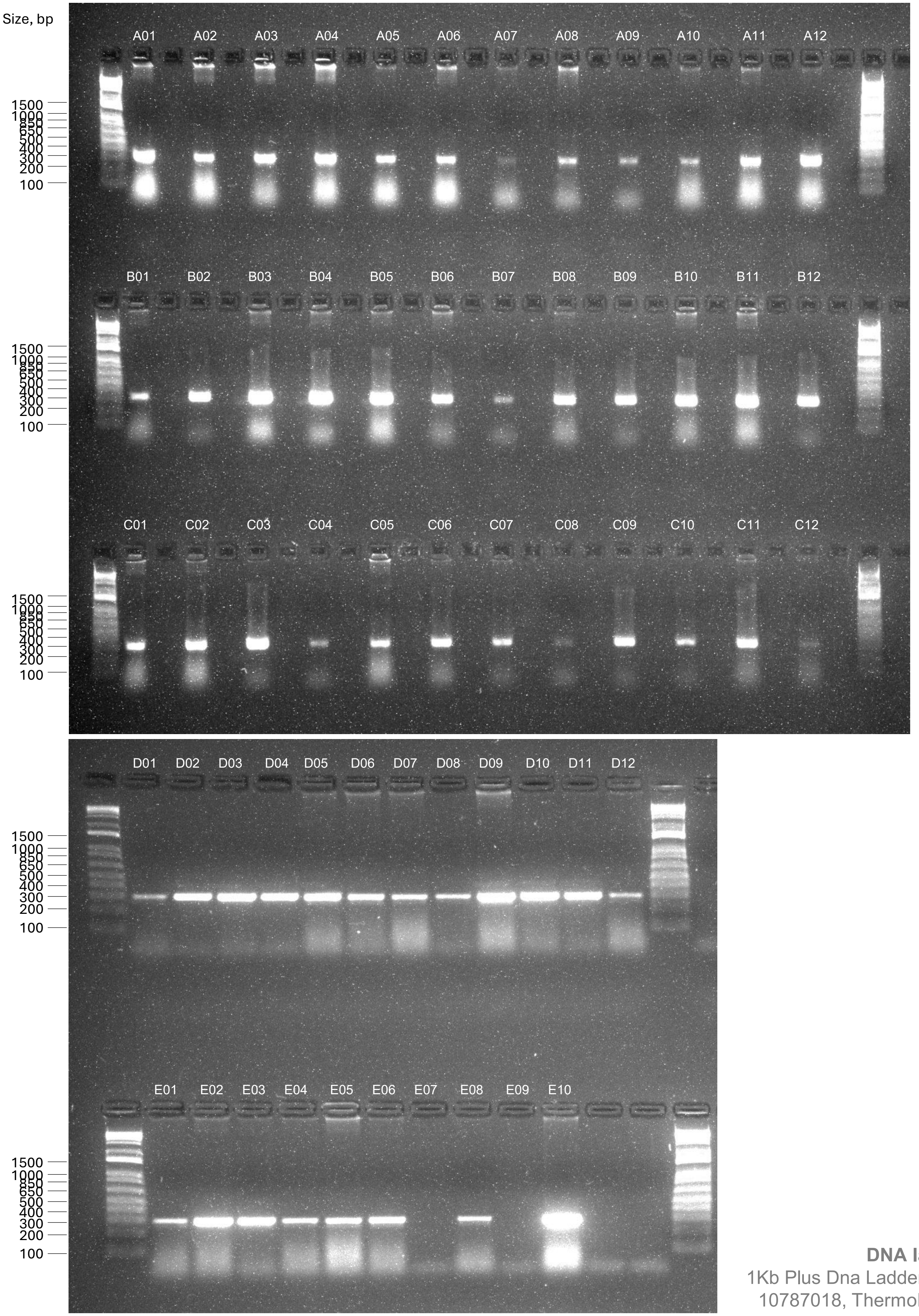

DNA ladder
1Kb Plus Dna Ladder, Ref. 10787018, ThermoFisher

(B) Diagnostic PCR amplification of target gene for KO validation

Left lane, Mutant gDNA
Right lane, Parental gDNA (control)

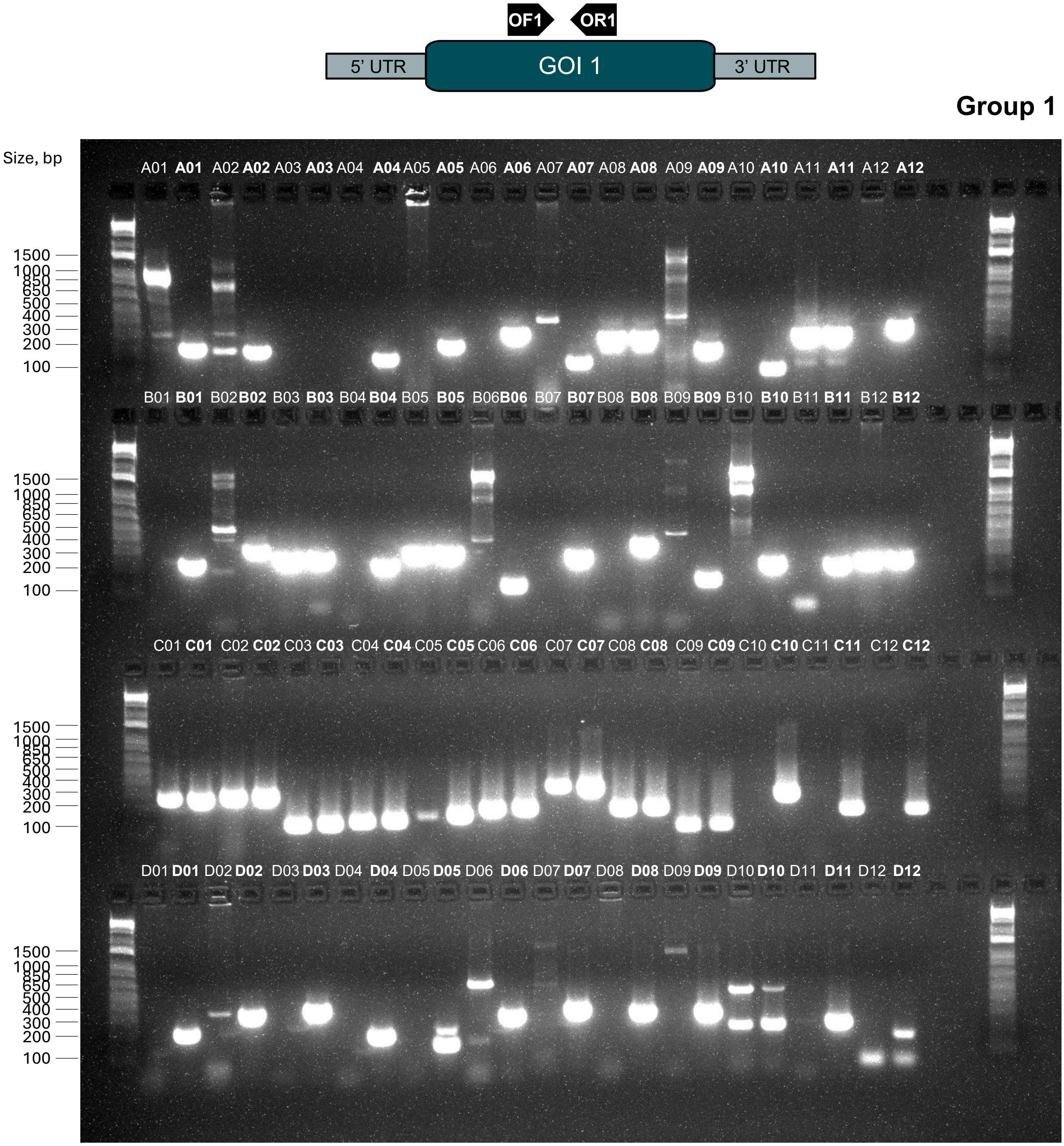

DNA ladder
1Kb Plus Dna Ladder, Ref. 10787018, ThermoFisher

Group 1

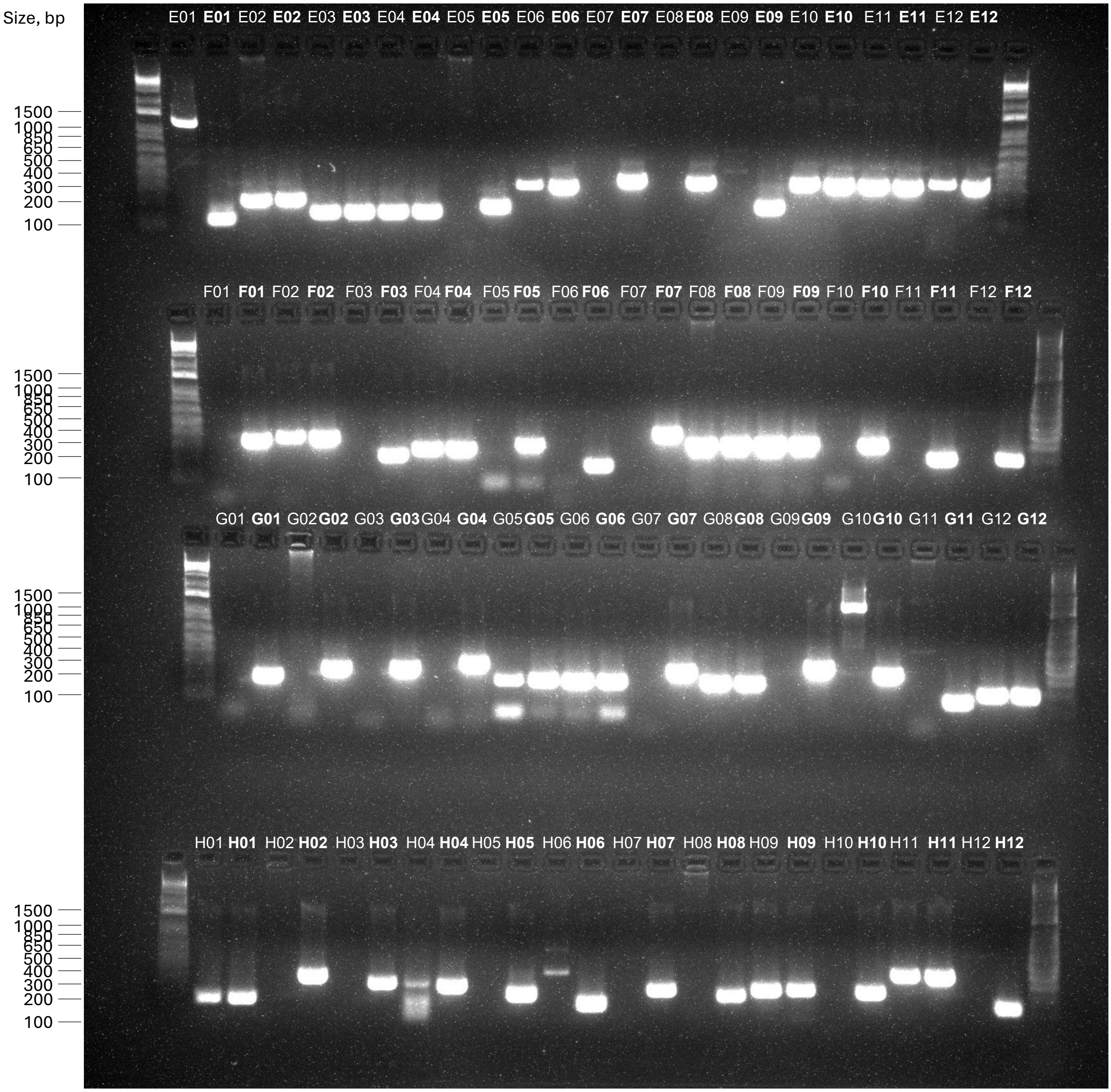

DNA ladder

1Kb Plus Dna Ladder, Ref. 10787018, ThermoFisher

Group 2

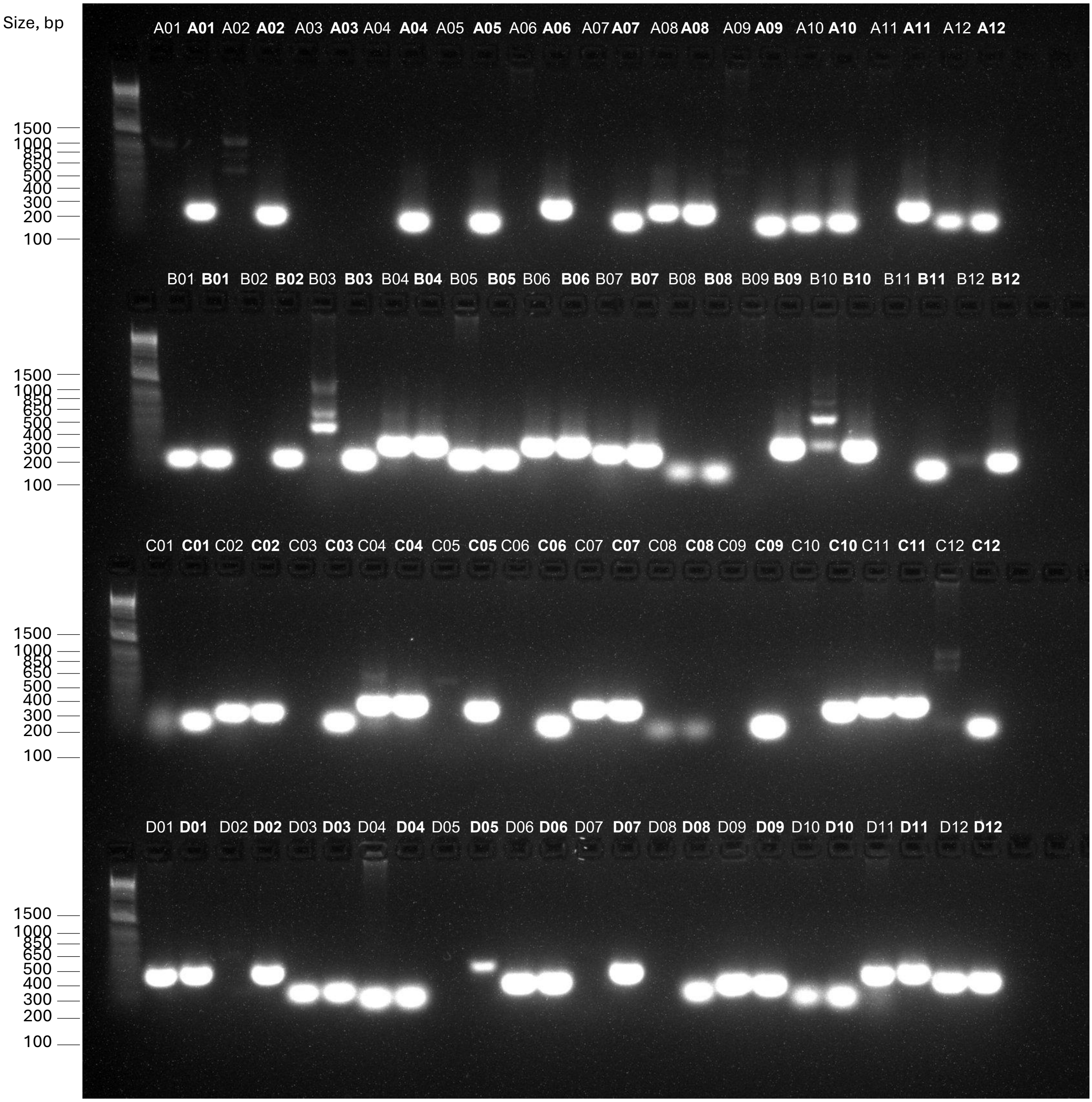

DNA ladder

1Kb Plus Dna Ladder, Ref. 10787018, ThermoFisher

Group 2

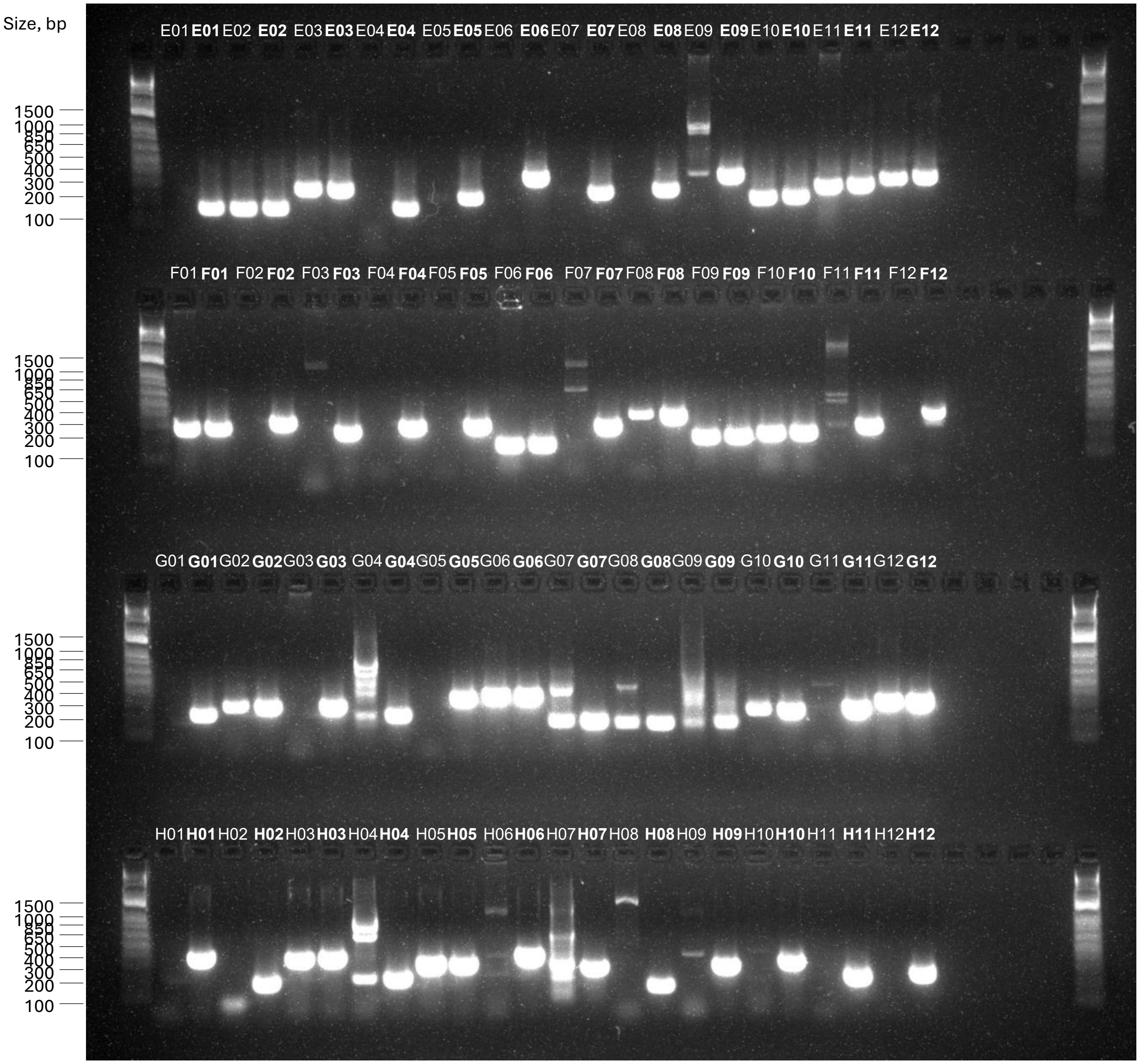

DNA ladder

1Kb Plus Dna Ladder, Ref. 10787018, ThermoFisher

Group 3

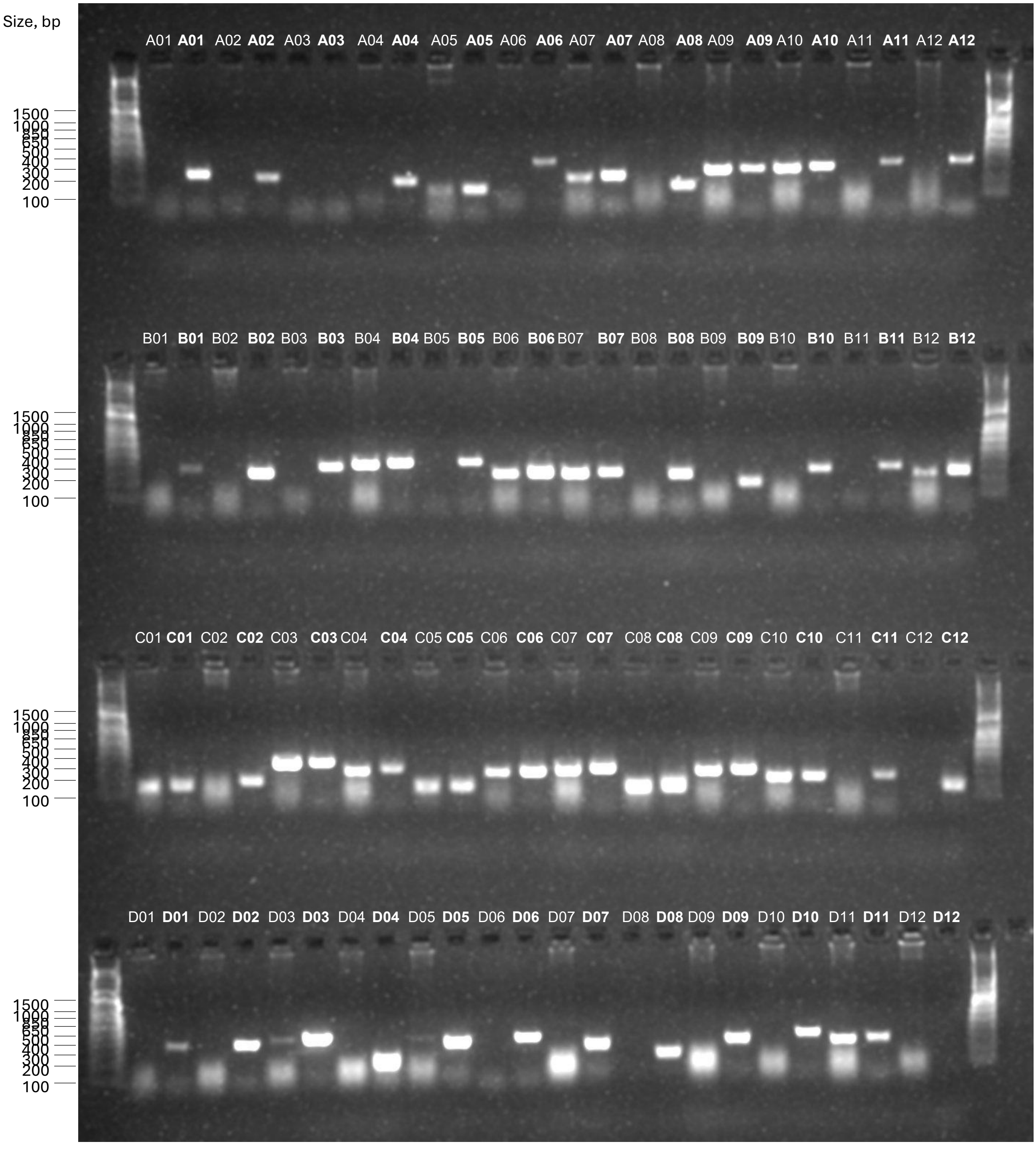

DNA ladder

1Kb Plus Dna Ladder, Ref. 10787018, ThermoFisher

Group 3

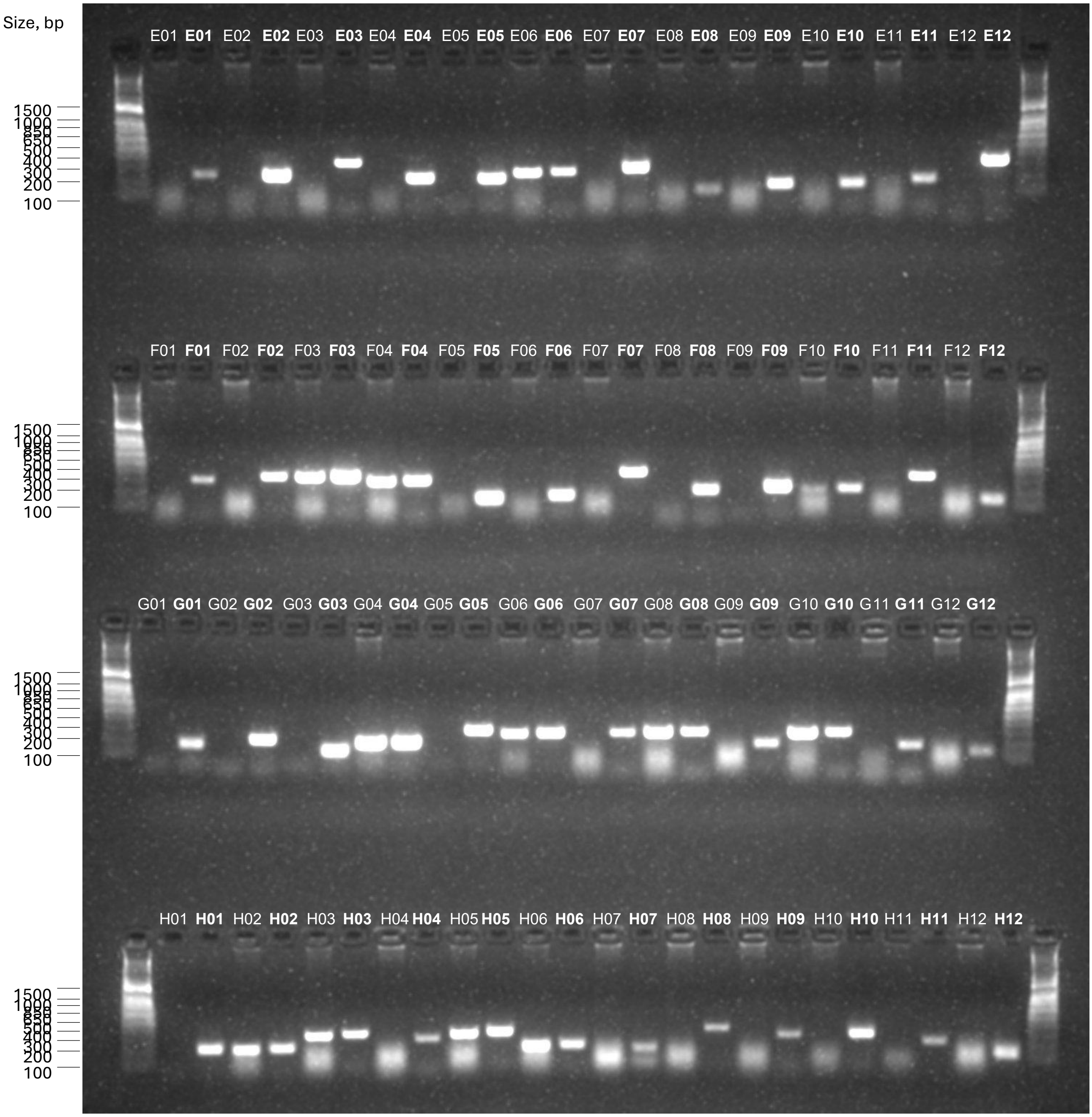

DNA ladder

1Kb Plus Dna Ladder, Ref. 10787018, ThermoFisher

Group 4

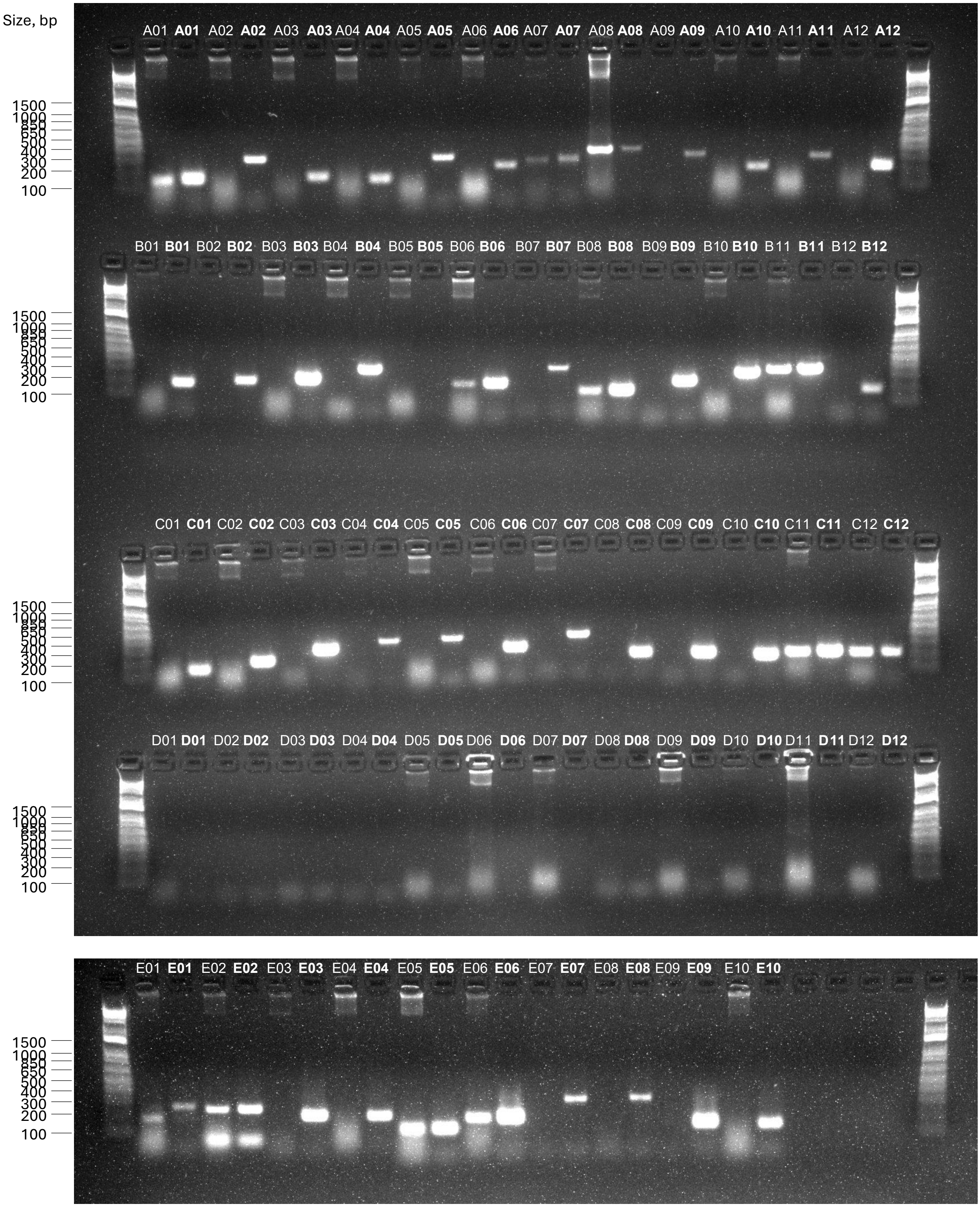

DNA ladder

1Kb Plus Dna Ladder, Ref. 10787018, ThermoFisher
