# Supplementary material for: Identification of transporters essential for survival of Leishmania promastigotes in the digestive tract of sand flies
Source: PLoS Pathog. 2026 Mar 16;22(3):e1014049. doi: 10.1371/journal.ppat.1014049 (PMC13004518; doi:10.1371/journal.ppat.1014049)
Supplement: Attachment — Submitted filename: Response to Reviewers.pdf [file ppat.1014049.s017.pdf]

REVIEWER COMMENTS FOR PPATHOGENS-D-25-01650
[IN BLUE] RESPONSES TO REVIEWERS COMMENTS

Part I – Summary

Combined response to "Part I":

We thank all 3 reviewers for their thoughtful and constructive feedback on our manuscript and recognition of the novelty of applying the transportome mutant library to sand fly infections and for pointing out the unique insights that the 34 genes exclusively associated with fitness costs in this context can provide. We are particularly appreciative for the reviewers' highlight of the data novelty, execution and manuscript quality, as well as the appropriate choice of control cell lines and validation approaches chosen in the present work. We also appreciate the attention to detail on the interpretation of the *in vivo* infection dynamics of the *Leishmania* promastigote cell line V-ATPase Subunit E knockout and the overall interest in a better understanding of the various emerging exciting aspects of the *Leishmania* V-ATPase and its biological context.

Several reviewers raised questions about the novelty of the findings and about the function of the V-ATPase in the insect vector. We would like to respond to these points here:

Novelty

This study reports several new findings that provide new insights into the molecular cell biology of *Leishmania* parasites in the sand fly vector. Specifically, we:

- Measured the fitness of >300 transporter gene deletion mutants in sand flies. This has never been done before.
- Identified 34 mutants that lost fitness only in flies and identified additional mutants where a loss of fitness is only detected in longer time courses *in vitro* (7 days in this study vs. 24 h of exponential growth in Albuquerque-Wendt *et al*, 2025)
- Show that the V-ATPase is essential for promastigotes in sand flies.
- Show that V-ATPase mutants failed to generate metacyclics *in vivo*. Additional data added to the revised manuscript show that promastigotes also fail to express the metacyclic marker *sherp in vitro*.
- Measured the bottleneck size in these experimental infections, providing an important reference for future bar-seq studies of vector-parasite interactions. These additional data were added to the revised manuscript.

Function of the V-ATPase

We have added a paragraph to the revised manuscript to explain that the V-ATPase is a highly conserved rotary proton pump whose principal function is the acidification of cell organelles. While its subunit composition and biochemical mechanism are well-characterised, its physiological functions are varied and context-dependent, and there is incomplete information about the physiological functions of V-ATPases in different kinetoplastid species and life cycle stages.

The results in this study indicate that V-ATPase is required for metacyclogenesis. We have previously shown that the *L. mexicana* V-ATPase is required in promastigotes for tolerance of an acidic external pH Albuquerque-Wendt *et al*, 2025. These different phenotypes are compatible with known physiological functions of V-ATPases in other cell types. A comprehensive series of follow-up experiments focusing on the cellular mechanisms impacted by *Leismania* V-ATPase activity, which are currently being led by us, exceed the scope of the current paper.

Responses to additional points raised by individual reviewer are below.

Reviewer #1: The results are clearly presented and interpreted and the manuscript is similarly well written.

This is the sandfly extension of a large study into the role of transporters in *Leishmania*. The global approach to generate and barcode CRISPR/Cas9 gene edited cell lines is powerful in that it allowed 300+ lines to be compared, simultaneously, in the same sandflies or culture flask. This removed much of the experimental

variation that would occur if they were worked on individually. The methods for culture and fly infection seem appropriate as do the methods of comparing the overall growth fitness and I appreciate that there needs a high throughput approach to analyze and interpret so many lines. I particularly liked the inclusion of reference mutant lines, based on their well characterized role for parasite colonization, to assess the robustness of the approach, particularly between the pools of mutant.

However, what I don't get a sense of is the how these gene disruptions, even the one that was singled out, the V-ATPase Subunit E, work in the vector.

Response: The knockout of Subunit E was chosen as a V-ATPase knockout representative, as we previously (Albuquerque-Wendt *et al*, 2025) observed that for most subunits of this protein complex, the mutants presented similar barcode trajectories in the sense that, their barcode proportions "crash" over time in a similar manner (both *in vivo*, and in later stages of *in vitro* grown promastigote cultures). We interpret these data to indicate that most subunits are required to assemble a fully functional V-ATPase. As so, we decided that in-depth characterisation of a single subunit knockout, should provide sufficient insights to the role of this multi protein complex.

This is partly hampered by the lack of a sampling point shortly after blood defecation. This is an important bottleneck in the *Leishmania*-sandfly interaction where those parasites that survive have the opportunity to colonize the rest of the sandfly and complete their development. As such, this would be a good comparator for both the day 2 and day 9 infections. I am not expecting the authors to revisit their experiments but I think this needs to be discussed in the context of their study. Despite this the authors manage to identify many new transporters that are beneficial or detrimental to sandfly infection.

Response: We do recognise that our experimental approach cannot distinguish whether mutants that failed to colonise the sand fly at 9 PBM, did so because they were eliminated with defecated blood remnants, or because they survived defecation but had other defects. Furthermore, since we took whole infected flies during our experiments, this study is also limited by the lack of distinction of where mutants were located in the sand fly's digestive tract. There may be mutants that have no replication defects (indistinguishable barcode counts) but are restricted to the posterior tract of the sand fly and fail to migrate to the anterior regions of the vector.
To gain more insight into the parasite's location within the fly (including ability to attach/resist loss by defecation) will require extensive follow-up work where the status of flies can be monitored and flies are dissected individually.

We addressed these limitations of our study in the revised discussion. Despite these limitations, our data highlights transporters needed to survive within the insect vector, regardless of when and where.

Reviewer #2: Sadlova and colleagues present the results of a bar-seq knock-out screen of Leishmania mexicana transporter genes and tandem gene arrays. The authors perform in vitro passage of promastigote cultures to assess the competitive fitness of the lines and then use these lines to infect Lutzomyia longipalpis sand flies. The fitness scores from this experiment identify 34 lines that are exclusively compromised in sand fly infections. The Vacuolar H+ ATPAse (V-ATPase) is singled out for validation and was identified as one of the transporters essential for parasite persistence and differentiation in the fly.

Overall the work is technically robust, and yields novel insight into transporters that are required for normal relative fitness of promastigote forms in the sand fly.
However, this is tempered by the work being incremental in nature and lacking mechanistic insight into the reason why V-ATPase is required in the sand fly but not in vitro. As a previous iteration of this library (by the same research team) has already been screened in bar-seq lifecycle profiling (albeit without the sand fly stage) the inclusion of a sand fly model is welcome, but not fully exploited here to yield substantial interest to the broader community of researchers studying host-pathogen interactions.

Strengths

Application of the sand fly model to the transportome KO library - novel insight into which transporters are important in this system.
Attempts to delete tandem gene arrays of various transporters.
Reproducibility of previous KO screens & phenotypes could be assessed.
Addback study to confirm V-ATPAse phenotype.

Weaknesses
Many of the KO phenotypes in vitro are already determined from previous work.
The 34 genes giving essential/important fitness defects in the sand fly are not explored in detail when these are the key insights arising from the study.
Addback of V-ATPase V1E could be used as a platform for functional genetics to explore the complex in detail but isn't.

Novelty
The researchers previously generated a barcoded library of transporter KO strains (TransLeish Project) which were profiled in vitro and in macrophage infections - therefore the in vitro aspect of the current manuscript is not really that novel. The reason for generation of a new library of strains is not explained in the current manuscript - it would be helpful to elaborate on this. It would also be useful to show the correlation between the results of the TransLeish screen and this screen, even at the level of ability to make a knockout. It seems that the authors were able to generate 46 extra mutants in this attempt compared to the last one, can they speculate on why that may be.

Response: This new library was generated for 3 main reasons; it allowed the inclusion of additional genes and gene arrays, and the generation of smaller sub-libraries to infect flies, as well as to organise sub-libraries by transporter gene families. We have now clarified this in the text and added the calculations of bottleneck size calculated from the barcode counts resulting from infections of sandflies with pooled libraries of different sizes.
A detailed correlation between the present results and those reported in Albuquerque-Wendt et al 2025, are available in Supplementary table 3, column K and L. For clarity name of cell L2 was changed from "Genotype in T1.0" to "Genotype reported in Albuquerque-Wendt et al 2025, PMID: 39747086."

On the point of novelty, we refer to our combined response above.

The application of this KO library to Sand Fly infections provides the element of novelty in the manuscript and the 34 genes associated with fitness cost exclusively in the sand fly can now provide insight into the unique aspects of this interaction, but this is left unexplored.

V-ATPase is defined as important in vivo but not in in vitro culture, an addback is performed of the V1E subunit KO mutant to validate this is a specific phenotype. However, there is no exploration of the protein complex function, for example using point mutations in V1E to explore the subunit molecular function (which the authors don't mention anyway). Most of the other descriptive characterisation of this complex was performed in a previous paper by the same team (localisation etc). The individual stresses which compromise the V1E are not explored with in vitro models using these mutants.

Response: We refer to our combined response to the questions about V-ATPase function above.

Reviewer #3: The study by Sadlova et al. investigated which transporter proteins are essential for Leishmania promastigotes. Using a library of over 300 Leishmania mutants, the researchers tested their ability to grow in laboratory conditions and to develop in the digestive tract of a sand fly. They reported that 34 transporter genes being of relevance for parasite survival inside the sand fly, and that the V-ATPase is especially important. The study is ambitious and generally well-executed with comprehensive molecular assessment.
The study lacks, however, important biological contextualization and some technical points must be clarified.

Response: We hope the added information about V-ATPase function, outlined in the combined response above, provides biological contextualization.

We refer to our clarification of technical points in Parts II and III.

Part II – Major Issues: Key Experiments Required for Acceptance

Reviewer #1: An interesting result of this paper was the enlarged phenotype of the nectomonad stage and diminished metacyclogenesis of the V-ATPase Subunit E knockout line but these were not worked on beyond observation. Perhaps this could be extended by looking for the effect of this mutation on metacyclogenesis in vitro; the ultrastructure (particularly, the volume or surface area of the mitochondrion) of the nectomonad cells; or see if this result can be replicated for nectomonads in vitro by subjecting them to stressors experienced in sandflies, such as pH or osmolarity changes, exposure to blood digestion products or lack of nutrients? Collectively, this would help begin to understand the role of this transporter for Leishmania.

Response: We thank the reviewer for this excellent suggestion, which led us to examine the V-ATPase V1E KO, addback and parental cell lines for the expression of the metacyclic marker *sherp* (Giraud *et al*, 2019; PMID: 30854476) during *in vitro* culture. The results show that the KO is deficient in *sherp* expression, corroborating the *in vivo* result: loss of V-ATPAse impairs metacyclogenesis. We have added this new data to the revised manuscript.

As noted above (Part I), the V-ATPase is likely to serve a variety of physiological roles, including acidification of acidocalcisomes and regulatory functions in the endo-lysosomal pathway (references provided in the revised manuscript), adaptation to acidic external pH (Albuquerque-Wendt et al., 2025) and facilitating metacyclogenesis (this study).

The cellular mechanisms impacted by *the* V-ATPase activity merit in-depth characterisation in a series of experiments which go beyond the scope of the current paper.

Reviewer #2: The authors have presented some solid descriptive biology, but I don't believe there is enough new, transformative, or mechanistic information to justify publishing in PLoS Pathogens; suggest manuscript is submitted elsewhere, for example PloS NTDs.

Specifically, several Leishmania bar-seq screens have been published in PLoS Pathogens which include profiling in vitro and in murine infections, but have also been followed up with detailed biochemical assessment of hit proteins , inducible deletion of essential proteins, and/or immunoprecipitation of novel protein interactions (Burge et al 2020, Damianou 2020). The validation of a single KO strain (V1E) by addback restoration, without any further functional genetics, biochemical or cellular exploration does not add much to the existing knowledge on this protein complex. If another, novel transporter had been chosen and characterised the manuscript would have been greatly strengthened.

Response: We thank reviewer #2 for highlighting the related *Leishmania* bar-seq studies (Burge *et al*, 2020 and Damianou *et al*, 2020) and agree that these works have significantly advanced the field by combining genetic screening with detailed mechanistic follow-up. We would like to note, however, that our study differs in several important respects. First, neither of these previous studies included the sand fly vector, which presents a more complex and variable environment than *in vitro* culture, and one that is central to the parasite life cycle and different from the environments encountered during mammalian infection. There have been only two previous bar-seq studies in sand flies, a screen of *L. mexicana* kinase deletions (Baker *et al*, 2021) and a screen of flagellar mutants (Beneke *et al*, 2019). Our work extends the bar-seq approach into the vector stage with a library that contains more mutants than the two other studies together, thus expanding the dataset on *Leishmania* phenotypes in sand flies significantly. Mutant screens in sandflies are technically very challenging. To address this under-appreciated point and facilitate future studies, we have added valuable additional data

to the revised manuscript: a calculation of the bottleneck size experienced by promastigote populations in the *in vitro* infection assays.

Perhaps adding experiments to define the individual stress or combination of stresses that compromise the V1E null mutant could be added to flesh out the biological insight of the work. Or if the authors have characterisation data of a novel transporter identified to be important in vivo it could be included to make it align better with PLoS Pathogens criteria for publication.

Response: We appreciate the reviewer's point and agree that many transporters remain poorly characterized. Our data identifies a relatively small number of transporters that appear to be crucial specifically for successful persistence in sand flies, out of a large cohort of ~300 genes. Characterizing novel transporters in detail is inherently challenging, as most lack defined substrates or clear functional annotations, thus necessitating a substantial program of additional biochemical characterisation to understand their substrate-specificity and substantial investment in additional sand fly infection experiments to study their function in the parasite life cycle. We believe this global dataset in itself makes a significant contribution to the field, and the demonstration that the V-ATPase is not only essential for infection of macrophages and mice (Albuquerque-Wendt et al., 2025) but also required for persistence in sand flies and metacyclogenesis adds mechanistic information.

We agree that the specific physiological functions of the V-ATPase in particular merit further in-depth characterisation and we refer to our responses to Reviewer I and our response in Part I, above.

Reviewer #3: 1) Line129: The authors state that promastigotes undergo terminal differentiation. This terminology should be avoided as it doesn't fit Leishmania life cycle. Inside the sandfly it has been shown that metacyclics dedifferentiate and regain ability to multiply after complete morphological reshaping. Beyond that, transmitted promastigotes will not remain as is. They will transform into amastigotes. "Terminal differentiation" imply permanently exiting the cell cycle and losing its ability to divide.

Response: We have amended the revised manuscript to take these valid points on board.

2) Figure 2: The variation in nutrient stress and other biotic and abiotic stochastic events is always present in the promastigote cycle. It will be very informative for this work to see a trasnportome actually working on a variable environment. Have you restarted the culture from 144hr time point? Also, 199 media, although widely used for Leishmania, is a media developed for fibroblasts. If using insect cell media, 30% or less of essentiality is still sustained?

Response: In this study we compared the fitness of mutants *in vitro* in M199 medium and *in vivo* in sand flies. The results show that fitness scores, overall, show a strong correlation between the two conditions. This means that, the M199 culture reproduces many of the conditions the promastigotes encounter in the sand flies, at least insofar as they concern the need for specific transporters. We have stressed in our revised manuscript that even in the relatively constant and glucose-rich *in vitro* cultures, the parasites were exposed to some variation as a result of population growth. We agree these data provide the basis for many interesting future experiments to test the effect of environmental changes (depletion of specific nutrients and growth factors, molecules accumulating in dense populations, changes to pH and temperature etc.) in a controlled and physiologically relevant setting.

3) How does the gain/loss of fitness of the mutant lines behave under multiple blood meals? Other authors have shown how important this constant change in the environment is crucial for vector competence. Beyond that, it's a certain event to happen in the sand fly biology and the course of promastigotes cycle. Authors should evaluate its effect on mutants' fitness.

Response: We appreciate the reviewer's point regarding the impact of multiple blood meals on parasite development and vector competence. We agree that repeated blood feeding represents a biologically relevant

scenario for *Leishmania* transmission, and that transporter mutants may display altered fitness under these conditions. We have now included a statement in the Discussion to emphasize this limitation.

Part III – Minor Issues: Editorial and Data Presentation Modifications

Reviewer #1: Could the authors clarify from the methods that '3 to 9 guts' were sampled at day 2 PBM? If so, do they think this is large enough to accurately determine the proportion of infected flies, the location of the infection and infection intensity? Was this the same for day 9 PBM, as this information is missing from the methods or the figure legends?

Response: In these types of experiments, evaluation of a small number of sand flies for qualitative inspection of infection intensity is routine. We work under the assumption that if the first 1-2 sand flies observed are heavily infected, most likely, the majority of the population is sufficiently infected to allow the screen to proceed. We recognise, though that the inability to sacrifice a larger amount of sand flies to accurately determine infection intensity, introduces the risk that a smaller number of flies, than those assumed, is in fact infected. This is an inherent limitation of this pooled approach that one would only be able to overcome if each sand fly is evaluated independently, which for large screens such as the ones here reported, is technically impractical. We further completed *Supplementary Table 8* with the detailed information on how many flies were evaluated per sample and their estimated infection intensity.

Reviewer #2: The number of sandflies used is a bit unclear - what was the number of fed flies used for DNA extraction per each sub-pool replicate of the bar-seq screen? Can this be detailed in the figure legend?

Response: We thank reviewer #2 for their attention to detail, we have added this detailed information to *Supplementary Table 8*.

Did the authors perform any power calculations to determine the minimum effect size that could be reliably determined by this assay type and the number of flies/replicates used?

Response: We refer to our combined response as well as our response in Part II, above.

For the in vitro growth were the selective antibiotics applied to the culture? If they were not does this influence the loss of barcodes from "refractory to deletion" strains due to the mosaic aneuploidy of Leishmania leading to loss of heterozygosity ? Thus delinking fitness of the barcoded strain to the amount of barcode?

Response: The promastigotes were cultured without selection drugs during the growth assay. The assay measures the barcode abundance in the population and how it changes over time, relative to the other barcodes. We included the cell lines designated as "incomplete deletions" since heterozygous knockout mutants may show a phenotype, even if complete loss of gene function is lethal. We refrained from drawing firm conclusions about individual phenotypes of "incomplete deletion" mutants, conscious that it would require examination of cell lines individually to track gene copy number over time, and establish how it relates to phenotype.

The manuscipt discusses "arrayed CRISPR/Cas9 mutant libraries" (the technique) and also "array mutants" (when talking about tandem gene arrays), and when the latter were introduced there were a couple of confusing instances; I would suggest that when referring to the tandem gene arrays this is explicit in every instance.

Response: We have amended the revised manuscript to avoid this confusion.

Is CRISPR a relevant term given that the authors are not using clustered regularly interspersed palindromic repeats in their system? Would Cas9-mediated homology-directed repair be more accurate?

Response: We use the term in the way it has become standard use in the literature.

Reviewer #3: 4) Figure 1 does not add much info to the manuscript. Part A is sufficient to tell the message and part B is too noisy for a main figure. I suggest you keep only A or move all to supplementary.

Response: We moved Figure 1 to Supplementary Figures.

5) Figure 4G: Manuscript will benefit from display of images from the parasites.

Response: We agree and added images of the parasites to new Figure 4.

6) Lines 384-396: This is good discussion. However, the authors will benefit by adding to this the fact that in vitro more than the correctly pointed is “not happening”. Many parasite stages just don’t appear in culture. Considering transporters, lacking in culture stages that will be present inside the vector may just lead to unrealistic loss/gain of fitness.

Response: We agree that *in vitro* cultures do not replicate the temporal and spatial dynamics of emergence of different morphotypes. This is the reason why we believe our data reporting on the fitness of >300 different *Leishmania* mutants in the sand fly is an important contribution to the literature and we hope an useful starting point for further *in vivo* studies.
